# Correlates of HIV treatment adherence self-efficacy among adolescents and young adults living with HIV in southwestern Uganda

**DOI:** 10.1371/journal.pgph.0003600

**Published:** 2024-09-04

**Authors:** Scholastic Ashaba, Charles Baguma, Patricia Tushemereirwe, Denis Nansera, Samuel Maling, Brian C. Zanon, Alexander C. Tsai

**Affiliations:** 1 Department of Psychiatry, Mbarara University of Science and Technology, Mbarara, Uganda; 2 Global Health Collaborative, Mbarara University of Science and Technology, Mbarara, Uganda; 3 Department of Pediatrics, Mbarara University of Science and Technology, Mbarara, Uganda; 4 Department of Pediatric Infectious Diseases, Emory University School of Medicine, Atlanta, Georgia, United States of America; 5 Department of Pediatric Infectious Diseases, Children’s Healthcare of Atlanta, Goergia, United States of America; 6 Department of Pediatric Infectious, Emory University Rollins School of Public Health, Atlanta, Georgia, United States of America; 7 Department of Psychiatry, Center for Global Health and Mongan Institute, Massachusetts General Hospital, Boston, Massachusetts, United States of America; 8 Department of Psychiatry, Harvard Medical School, Boston, Massachusetts, United States of America; The Ohio State University, UNITED STATES OF AMERICA

## Abstract

Adherence to antiretroviral therapy (ART) among adolescents and young adults living with HIV (AYLHIV) in sub-Saharan Africa is sub-optimal compared to younger children and older adults. Adherence self-efficacy is one of the intrapersonal factors most strongly correlated with ART adherence. The role of adherence self-efficacy in ART adherence among AYLHIV is not well studied in Uganda. We enrolled 300 AYLHIV between October and December 2021 from an HIV clinic in southwestern Uganda. We collected information on adherence self-efficacy, HIV stigma, depression, self-management, and social skills. We used linear regression to estimate the association between adherence self-efficacy and the covariates of interest. At multivariable adjustment self-management (b = 0.29, 95% CI 0.23–0.35, p<0.001) and social skills (b = 0.16, 95% CI 0.08–0.24; p<0.001) were statistically significantly associated with adherence self-efficacy. The findings imply that interventions directed at enhancing self-management and social skills in AYLHIV may increase adherence self-efficacy and, potentially, HIV outcomes among AYLHIV.

## Introduction

Although availability of and access to antiretroviral therapy has enabled children born with HIV to survive into adolescence and young adulthood [1, 2], HIV remains a leading cause of mortality among adolescents in sub Saharan Africa [3, 4]. In Uganda, there are 170,000 adolescents living with HIV, constituting 12% of the people living with HIV nationwide [5]. HIV treatment among adolescents and young adults is characterized by poor adherence to HIV antiretroviral therapy (ART) and high risk of viral failure, loss to follow up, and overall mortality [6–10]. Compared to older adults and younger children, adolescents and youth living with HIV (AYLHIV) exhibit worse outcomes across the continuum of treatment related behaviors [11, 12], and in Uganda specifically [13, 14]. Factors associated with ART adherence among AYLHIV include HIV stigma, lack of social support, medication side effects, lack of HIV status disclosure and depression [15–19].

Self-efficacy is a primary facilitator of ART adherence among adults living with HIV [20, 21]. Self-efficacy is defined, in the context of HIV treatment, as the belief in one’s ability to effectively consistently take medications, attend clinic appointments and follow treatment guidelines in general [22]. It is considered to be essential for establishing and maintaining specific health behaviors, which explains its effect on ART adherence [23, 24], and has been identified as one of the major intrapersonal factors governing ART adherence [25, 26]. Associations between adherence self-efficacy, treatment adherence, and viral suppression have been documented among people living with HIV including both adults and adolescents [24, 25, 27–29]. Self-efficacy provides a buffer against the barriers faced by people living with HIV, allowing them to persist with treatment despite encountering such challenges [25, 30–32]. In Uganda, the role of self-efficacy in explaining treatment adherence among AYLHIV has not been studied. Understanding drivers of adherence self-efficacy among AYLHIV will inform development of appropriate interventions to improve ART adherence in this population, especially as they grow towards the eventual transition to adult HIV care (when there will be a greater expectation that they manage their care independently [33, 34]). This analysis focused on identifying correlates of adherence self-efficacy among AYLHIV in rural southwestern Uganda.

## Materials and methods

### Study setting and participants

Participants were recruited from the HIV clinic at Mbarara Regional Referral Hospital (MRRH). MRRH is located in Mbarara city, in southwestern Uganda, approximately 270 km from the capital city, Kampala. The population of Mbarara city is 195,013 according to the last census conducted by the Uganda Bureau of Statistics [35]. Most of the people who attend the HIV clinic reside in nearby communities and earn their livelihood through subsistence farming, animal husbandry, and small scale trading despite acknowledged challenges of water and food insecurity [36, 37]. According to estimates, the prevalence of HIV in Mbarara District is 13%, which is higher than the 5.8% prevalence nationally [38]. The HIV clinic at MRRH offers a variety of services to people with HIV, including ART, viral testing, and adherence counselling.

We recruited adolescents and young adults living with perinatally acquired HIV (AYLPHIV) aged 15–24 years, who were fully aware of their HIV status (i.e., through disclosure to them by their parents or guardians), living within 60 km of the clinic and able to provide written informed assent and/or consent. We excluded AYLPHIV who could not tolerate the length of the interview, those who were not aware of their HIV status (i.e., because their parents or guardians had not disclosed to them their status), and those who were too cognitively impaired to participate in the interview as assessed by the attending clinician in consultation with a licenced Ugandan psychiatrist.

### Enrolment procedure

Participants were recruited consecutively as they came to the HIV clinic for their regular clinical reviews between October and December 2021. A research assistant fluent in both Runyankore and English conducted face to face interviews. Those who met the eligibility criteria were approached by the research assistant who introduced the study and requested their participation. The research assistant administered a questionnaire to those who provided written informed assent or consent to participate.

### Study measures

#### Adherence self-efficacy

Adherence self-efficacy was assessed using the 12-item HIV treatment adherence self-efficacy scale (HIV-ASES) [25]. The HIV-ASES assesses confidence in carrying out medication related behaviors including adhering to treatment plans, following the treatment regimen, nutritious food, and engaging in exercise, despite the challenges they are likely to face. Sample items include “can stick to your treatment plan even when side effects begin to interfere with daily activities” and “can continue with your treatment even if doing so interferes with your daily activities.” The scale has been translated for use among adults living with HIV in Uganda [39, 40]. Responses for each item were scored on a modified scale ranging from 1 (cannot do at all) to 3 (completely certain can do). We calculated the total score by summing across the items such that the minimum possible score was 12 and the maximum was 36. The scale had a Cronbach’s alpha of 0.96 in this study.

#### Depression

Depression was assessed using a 20-item depression scale that was developed in Mbarara, southwestern Uganda for use among adolescents living with HIV [41]. The scale captures both affective and cognitive symptoms of depression. Sample questions include “In the last two weeks, how often have you felt hopeless about the future?” and “In the last two weeks, how often did you feel you had no peace?” Each item is scored on a 4-point Likert-type scale, ranging from 0 (not at all) to 3 (all the time). The minimum possible score is 0 and the maximum possible score is 60. In this study the scale had a Cronbach’s alpha of 0.92.

#### HIV stigma

HIV stigma was assessed using the 10-item HIV stigma scale for adolescents living with HIV [42]. The scale was developed among adolescents living with HIV in South Africa and consists of 5 items measuring internalized stigma, 2 items measuring anticipated stigma, and 3 items measuring enacted HIV stigma. Each item is scored on a 3-point Likert-type scale, ranging from 0 (never) to 2 (most of the time) with a minimum possible score of 0 and a maximum possible score of 20. In this study the scale had an overall Cronbach’s alpha of 0.78.

#### Self-management skills

Self-management skills were assessed using the 21-item self-management skills assessment guide, a comprehensive self-rated questionnaire for measuring medical self-management and readiness to transition in youth that was developed in Canada [43]. Each item is scored on a 5-point Likert scale ranging from 1 (strongly disagree) to 5 (strongly agree) with a minimum possible score of 21 and a maximum possible score of 105. In this study the scale had a Cronbach’s alpha of 0.89.

#### Social skills

To assess social skills, we administered the 25-item social skills assessment scale for adolescents. The items were adapted from Goldstein and McGinnis [44]. Each question is scored on a 3-point Likert-type scale ranging from 1 (almost never) to 3 (almost always). The possible minimum score is 25 and the maximum score is 75. Sample questions include “I am good at finding fair ways to solve problems” and “I ask questions about things I don’t understand.” In this study the scale had a Cronbach’s alpha of 0.92.

### Data analysis

Data were analysed using Stata version 17. We fit multivariable linear regression models with adherence self-efficacy specified as the dependent variable and HIV stigma, depression, self-management and social skills as the primary explanatory variables of interest. Regression models also adjusted for age, sex, level of education, primary caregiver, age when started ART, duration on ART and marital status.

### Ethical considerations

The study was approved by the Research Ethics Committee of the Mbarara University of Science and Technology (#20/08-19) and the Partners Human Research Committee (#2019P003451). The study also received clearance from Uganda National Council for Science and Technology (#HS512ES) and the Office of the President. In accordance with recommendations from the Uganda National Council for Science and Technology [45], emancipated minors—defined as adolescents under the age of 18 who are either pregnant, have a child, or are responsible for their own livelihood—and empowered adolescents—defined as adolescents under the age of 18 who are empowered to take responsibility for their own health—were permitted to give written informed consent without the involvement of their caregivers. All participants received 25,000 Ugandan Shillings (approximately 7 U.S. dollars at the time the study was conducted) to reimburse them for transportation to the clinic/study site.

## Results

We enrolled 300 participants. The mean age was 19.1 years (standard deviation [SD] = 2.81; range, 15–24 years), and more than half (57%) were girls and young women. Duration on ART was 15 years (SD = 4.7) and most (87%) had not yet attempted to transition to adult HIV care. The mean adherence self-efficacy score was 29.2 (SD = 5.93) (Table 1).

**Table 1:** Summary characteristics of the study participants (N = 300).

Variables	Mean (SD) or n	%
Age, years	19.1 (2.8)	
*Sex*		
Female	171	57%
Male	129	43%
Age when started ART, years	4.5 (4.7)	
Duration on ART, years	15 (4.7)	
*Marital status*		
Single	284	95%
Married	16	5%
*Education level*		
Primary	79	26%
Secondary	180	60%
Above secondary	41	14%
*Main caregiver*		
Father alone	19	6%
Mother alone	104	35%
Both parents	70	23%
Grandparents	26	9%
Other relative	81	27%
*Transition status*		
Have not yet transitioned	261	87%
Transitioned successfully	11	4%
Failed transition	28	9%
*HIV care status*		
Active in care	296	99%
Not active in care	4	1%
HIV stigma score	3.3 (3.2)	
Depression score	7.9 (8.6)	
Social skills score	59.7 (8.8)	
Self-management score	72.1 (11.8)	

On bivariate analysis, adherence self-efficacy had a statistically significant association with social skills and self-management but not with depression or HIV stigma. In a multivariable linear regression model, both retained a statistically significant association with adherence self-efficacy: self-management (b = 0.29, 95% CI 0.23–0.35; p<0.001) and social skills (b = 0.15, 95% CI 0.07–0.24; P<0.001) (Table 2).

**Table 2:** Correlates of adherence self-efficacy among the study participants.

	Bivariate		Multivariable	
Variable	b (95%CI)	p-value	b (95%CI)	p-value
Age	0.21 (-0.02 to 0.45)	0.08	-0.31 (-1.71 to 1.09)	0.66
Age when started ART (years)	0.13 (-0.01 to 0.27)	0.07	-0.17 (-1.66 to 1.32)	0.82
Duration on ART (years)	-0.07 (-0.22 to 0.07)	0.31	-0.14 (-1.54 to 1.26)	0.84
*Sex*				
Male	Ref	Ref		
Female	-0.14 (-1.50 to 1.22)	0.84	0.03 (-0.98 to 1.05)	0.95
*Caregiver*				
Father alone	Ref	Ref		
Mother alone	0.49 (-2.41 to 3.38)	0.74	1.09 (-1.07 to 3.26)	0.32
Both parents	0.78 (-2.22 to 3.78)	0.61	1.81 (-0.44 to 4.07)	0.12
Grandparents	0.74 (-2.764.24)	0.68	2.66 (0.047–5.28)	0.05
Other relatives	2.62 (-0.33 to 5.58)	0.08	2.23 (-0.10 to 4.57)	0.06
*Education level*				
Primary	Ref	Ref		
Secondary	-0.61 (-2.16 to 0.94)	0.43	-2.45 (-3.73 to -1.16)	<0.001
Above secondary	3.06 (0.86 to 5.27)	<0.001	-0.62 (-2.57 to 1.33)	0.53
Depression	-0.03 (-0.11 to 0.04)	0.39	-0.02 (-0.09 to 0.04)	0.46
Self-management	0.32 (0.27–0.36)	<0.001	0.29 (0.23–0.35)	<0.001
Stigma	-0.07 (-0.28 to 0.14)	0.52	-0.02 (-0.19 to 0.16)	0.83
Social skills	0.34 (0.27–0.41)	<0.001	0.16 (0.08–0.24)	<0.001

Thus, a one-standard deviation difference in self-management was associated with a 3.37-point difference in adherence self-efficacy, or a 12% difference relative to the sample mean and 0.57 standard deviation units. Similarly, a one-standard deviation in social skills was associated with a 1.38-point difference in adherence self-efficacy (5% relative to the sample mean and 0.23 standard deviation units).

## Discussion

In this study of 300 AYLPHIV recruited from an HIV clinic in rural Uganda, we found that social skills and self-management were significantly associated with adherence self-efficacy. The association between self-management and adherence self-efficacy was both statistically significant and large in magnitude. It echoes findings of previous studies among adolescents and adults living with HIV in Ethiopia and South Africa [46, 47]. The estimated association is likely bidirectional [48, 49]. Self-management comprises motivation, understanding of the illness, confidence in one’s ability to manage the illness and access HIV services, and self-advocacy to seek the necessary support as one navigates HIV care—all of which constitute self-efficacy [50, 51]. Self-efficacy improves self-management behaviors, including adherence to ART [52–54]. Enhancing self-efficacy leads to changes in behaviour and improves people’s abilities to manage their illness [55] and has the potential to improve quality of life among people living with HIV [56].

The observed association between social skills and adherence self-efficacy found in our study was smaller in magnitude. It is consistent with the argument that good social skills are associated with a larger social network, which provides opportunities for verbal reassurance, peer learning, and positive reinforcement of adherence behaviour [57]. AYLHIV with high social skills may be more likely to receive financial assistance and other logistical support through their social connections, which may enable them to access HIV care and remain in care [58]. AYLHIV with good social skills may also be more likely to gain social acceptance and reciprocal support, empowerment and personal growth, all of which can increase a sense of self-efficacy [59], treatment adherence, and retention in care [60]. Good social skills have also been associated with patient-provider communication, which has been linked to medication adherence [61].

Contrary to our hypotheses, we did not find an association between HIV stigma and adherence self-efficacy. The finding of no association between HIV stigma and adherence self-efficacy is in agreement with findings of a study among adolescents living with HIV in Kenya [29] but is contrary to findings of previous studies among adults living with HIV [62, 63]. The studies that have reported an association between adherence self-efficacy and HIV stigma focused on enacted stigma and were conducted among adult populations [24, 62]. In one study of adolescents and young adults living with HIV, higher levels of self-efficacy were associated with low levels of internalized HIV stigma [64].

Our results should be interpreted in view of the following limitations. First, we enrolled participants from one HIV treatment facility and over a short period of time (3 months), hence the lack of diversity in terms of geographical location and clinic setting may limit the generalizability of the findings to other populations of AYLHIV in Uganda or other sub-Saharan African countries. Second, we only enrolled adolescents and young adults with perinatally acquired HIV, so our findings may not reflect personal beliefs about HIV treatment behaviours among adolescents and young adults with behaviorally acquired HIV. Third, the study was cross sectional in nature, hence we could not estimate the causal effect of self-management and social skills adherence self-efficacy. Last but not least, we left out other factors that might also be important in assisting in establishing a more comprehensive understanding of the factors influencing adherence self-efficacy among AYLHIV, such as socioeconomic status, access to healthcare services, and peer support networks.

## Conclusions

The findings imply that interventions directed at enhancing self-management and social skills in AYLPHIV might increase adherence self-efficacy, which may in turn promote adherence to ART.

## Supporting information

S1 Checklist Inclusivity in global research. (DOCX)
