# Correction: Correlates of HIV treatment adherence self-efficacy among adolescents and young adults living with HIV in southwestern Uganda

**DOI:** 10.1371/journal.pgph.0003874

**Published:** 2024-10-15

**Authors:** Scholastic Ashaba, Charles Baguma, Patricia Tushemereirwe, Denis Nansera, Samuel Maling, Brian C. Zanon, Alexander C. Tsai

There are errors in the funding statement. The correct funding statement is as follows: This research was supported by the Harvard University Center for AIDS Research (CFAR), an NIH funded program (P30AI060354), which is supported by the following NIH Co-Funding and Participating Institutes and Centers: NIAID, NCI, NICHD, NIDRCR, NHLBI, NID, NIMH, NIA, NIDDK, NINR, NIMHD, FIC, and OAR. The authors acknowledge salary support from NIH K43TW011929 (SA) and NIH K24DA061696 (ACT). The funders had no role in study design, data collection and analysis, decision to publish, or preparation of the manuscript.
